# Cost‐Effective and Time‐Saving Multiplex‐Beads Immunoassay Method to Detect Humoral Immune Responses Against Respiratory Infections

**DOI:** 10.1155/jimr/8000849

**Published:** 2026-08-26

**Authors:** Tarfa A. Altorki, Mohammad Abdullah Basabrain, Abdulaziz Kulthum, Maimonah Alghanmi, Ala A. Azhari, Sawsan Alamri, Aishah Ghazwani, Asem Alsharef, Wesam H. Abdulaal, Rahaf Y. Alhabbab, Mohamed A. Alfaleh, Anwar M. Hashem, Rowa Y. Alhabbab

**Affiliations:** ^1^ Department of Medical Laboratory Sciences, Faculty of Applied Medical Sciences, King Abdulaziz University, Jeddah 21589, Saudi Arabia, kau.edu.sa; ^2^ Vaccines and Immunotherapy Unit, King Fahd Medical Research Center, King Abdulaziz University, Jeddah 21589, Saudi Arabia, kau.edu.sa; ^3^ Department of Biochemistry, Faculty of Science, King Abdulaziz University, Jeddah, Saudi Arabia, kau.edu.sa; ^4^ Department of Clinical Microbiology and Immunology, Faculty of Medicine, King Abdulaziz University, Jeddah, Saudi Arabia, kau.edu.sa; ^5^ Department of Oral and Maxillofacial Surgery, King Fahad General Hospital, Jeddah, Saudi Arabia; ^6^ One Health Research and Innovation Center (OHRIC), King Faisal University, Al-Hofuf, Al-Ahsa, Saudi Arabia, kfu.edu.sa

**Keywords:** flowcytometry, IgG, IgM, MERS-CoV, multiplex-bead, SARS-CoV-2

## Abstract

The multiplex‐bead immunoassay is characterized by its ability to detect multiple analytes simultaneously within a short timeframe and with high precision. Consequently, it is an appealing tool for identifying humoral immune responses to various targets, particularly respiratory viral infections that pose a significant risk to public health. In this study, we optimize the assay by using minimal reagent volumes to reduce both costs and turnaround time without compromising assay accuracy. We have developed a multiplex‐bead immunoassay to detect humoral responses against Severe Acute Respiratory Syndrome COVID‐19 2 (SARS‐CoV‐2) and Middle East Respiratory Syndrome COVID‐19 (MERS‐CoV) on a platform that can be extended to include additional targets. To validate the performance of the developed assay, serum samples from MERS‐CoV‐ and SARS‐CoV‐2‐seropositive and seronegative individuals were evaluated and compared with the standard enzyme‐linked immunosorbent assay (ELISA) method. The results demonstrate that the developed multiplex‐bead immunoassay exhibits high performance, achieving 100% sensitivity and 95.4%–96.6% specificity, a detection limit comparable to ELISA, and a strong correlation with ELISA (*R* ^2^ = 0.91–0.98) in detecting humoral responses in the tested samples using minimal reagents. Overall, the developed immunoassay offers a rapid method for simultaneously detecting humoral responses for multiple analytes, with high sensitivity and specificity and lower costs than ELISA.

## 1. Introduction

Respiratory infections, especially viral infections, are among the most virulent diseases that can rapidly cause epidemics and pandemics. The rapid spread of viral infections among populations is the leading threat observed during the severe acute respiratory syndrome coronavirus 2 (SARS‐CoV‐2) pandemic. Some infected individuals can be asymptomatic and unknowingly contribute to disease spread [1–3]. Despite the Centers for Disease Control and Prevention (CDC) reporting a decline in COVID‐19 (Coronavirus disease 2019) cases, mainly due to vaccines, Middle East Respiratory Syndrome COVID‐19 (MERS‐CoV) remains a threat in the absence of vaccines, particularly in high‐risk areas (WHO, 2025). The proper diagnosis and medical intervention are crucial for patient survival and to control the spread of viral diseases. With most viral respiratory infections indistinguishable by common symptoms, it is vital to have a test to help overcome these issues. Although reverse‐transcriptase (RT)‐PCR is the standard method for detecting most respiratory infections, detecting viral antigens either with RT‐PCR or one of the available rapid tests is associated with some degree of false‐negative results due to the timing and the quality of the collected swab samples, mainly if it was during the declining stage of the viral load in the upper respiratory tract [4–7]. Li et al. [7] and colleagues showed that RT‐PCR is associated with about 20% false‐negative results, and they found that, among every 10 negative RT‐PCR results, two were confirmed true positives for COVID‐19. Therefore, antigen‐detecting tests should be accompanied by serological assays to obtain precise results [8].

Additionally, RT‐PCR requires a long turnaround time (≈2–10 h), which can be overcome by using point‐of‐care tests. However, both tests can only detect one antigen at a time. Therefore, it is essential to provide a test to identify asymptomatic, recovered, or patients with declining viral load for more than one target simultaneously. The immune status of patients against a given pathogen can be determined by measuring IgM and IgG antibodies, which reflect the stage of infection in an individual. The standard method for detecting individual humoral responses is the enzyme‐linked immunosorbent assay (ELISA); however, the main limitations of ELISA are the long turnaround time and the ability to measure only one analyte at a time. Thus, in this study, we aimed to develop a multiplex‐bead immunoassay to detect humoral responses against SARS‐CoV‐2 and MERS‐CoV in serum using flow cytometry, which is available in most, if not all, clinical laboratories and offers high sensitivity and specificity. Moreover, the developed assay has been fully established in‐house and refined through multiple stages to provide a low‐cost test that can also be extended to include additional targets while using only three fluorochromes. Multiplex‐bead immunoassay overcomes the limitations associated with ELISA, including the long time to perform the test and the ability to detect only one analyte at a time.

## 2. Materials and Methods

### 2.1. Study Design

The development of the in‐house multiplex‐bead immunoassay detecting IgM and IgG specific for the SARS‐CoV‐2 *Nucleocapsid* (N) protein and the MERS‐CoV *Spike* (S1) subunit was achieved through iterative steps. Assay performance was evaluated using sera collected from confirmed positive and negative individuals via ELISA and RT‐PCR. The data from the developed multiplex‐bead immunoassay were compared and validated against the standard method for detecting antibodies in serum, ELISA.

### 2.2. Ethics

All samples were anonymised and utilized in accordance with previously obtained ethical approvals from the Institutional Review Board at the Ministry of Health, Saudi Arabia (IRB Numbers: H‐02‐J‐002 and Project Number: 1367). Moreover, all participants have provided informed consent.

### 2.3. Participants

Samples used in this investigation were obtained from a previously conducted cross‐sectional study of healthcare workers in Jeddah, Saudi Arabia. The samples were collected between June 29 and August 10, 2020. They included a random selection of volunteers from three main referral hospitals in the area, along with staff working at five quarantine sites. All participants were over 17 years old. As for the MERS‐CoV and control samples, they were randomly collected from the western region of Saudi Arabia between 2011 and 2016. Specimens were collected carefully and treated cautiously as they were infectious and hazardous. Thus, all samples were handled in accordance with standard biosafety procedures. The samples were collected using yellow‐top collection tubes. After centrifugation, the separated serum was carefully collected into several Eppendorf tubes and stored at −80°C. It has been used in this study and in previously published work [8–10].

### 2.4. Recombinant SARS‐CoV‐2 N Protein and MERS‐CoV S1 Subunit Expression and Purification

Recombinant SARS‐CoV‐2 N protein was expressed in *Escherichia coli* BL21 (DE3) cells, as described previously [2, 3]. Meanwhile, the recombinant MERS‐CoV S1 subunit was expressed in ExpiCHO‐S cells using PEI‐mediated transfection [11]. The expressed proteins were purified using HisTrap Excel affinity chromatography. After loading the culture supernatant, wash buffer (20 mM sodium phosphate, 30 mM imidazole, 500 mM NaCl, pH 7.4) was used to wash the HisTrap Excel column (Cytiva, Marlborough, MA, US). The protein was then eluted using elution buffer (20 mM sodium phosphate, 500 mM imidazole, 500 mM NaCl, pH 7.4). The HiPrep 26/10 column (Cytiva, Marlborough, MA, US) was used to desalt the eluted proteins into phosphate‐buffered saline (PBS), and a 0.22 μm filter was used to filter the solution. Using a Nanodrop 1000 spectrophotometer and the computed extinction coefficient, the concentration of eluted protein was measured at 280 nm.

### 2.5. Indirect ELISA Detecting SARS‐CoV‐2 N Protein IgM and IgG Antibodies

ELISA was performed as previously published by our group [12]. Briefly, ELISA plates were coated with the purified SARS‐CoV‐2 recombinant N protein at 4 μg mL^−1^ and kept overnight at 4°C. Following washing and blocking the plates, the serum samples were added at 1:100 for 1 h at 37 °C. After incubating the plates for 1 h at 37°C with HRP‐conjugated antihuman IgM or IgG and washing, the 3,3^′^, 5,5^′^‐tetramethylbenzidine (TMB) substrate was added. A stop solution was used to stop the reaction (2M H_2_SO_4_). The absorbance was measured by Synergy 2 Multidetection Microplate Reader (BioTek, Winooski, VT) at 650 nm.

### 2.6. Indirect ELISA Detecting MERS‐CoV S1 Subunit IgM and IgG Antibodies

ELISA was performed as described in our previously published work [9]. We coated the ELISA plates with 2 g mL^−1^ MERS‐CoV recombinant S1 subunit overnight at 4°C in PBS. After washing and blocking the plates for 2 h, the serum samples were added at 1:100 dilution for 1 h. Then, the plates were washed, and HRP‐conjugated antihuman IgM or IgG was incubated for 1 h. Next, the TMB substrate was added, and the reaction was stopped with a stop solution (2M H_2_SO_4_). The absorbance was measured by Synergy 2 Multidetection Microplate Reader (BioTek, Winooski, VT) at 650 nm.

### 2.7. Production of Carboxyl Beads Conjugated to SARS‐CoV‐2 N Protein, and MESR‐CoV S1 Subunit

The carboxyl beads (BioLegend, UK) were conjugated according to the manufacturer’s instructions. The desired amount of beads was centrifuged, and the supernatant was replaced with a coupling buffer. After several washes with the coupling buffer, a solution of 50 mg/mL 5‐ethynyl‐2‐deoxycytidine (EDC) and N‐hydroxysulfosuccinimide (Sulfo‐NHS) was added, and the mixture was incubated for 20 min at room temperature (RT) on a rotary shaker at 1000 rpm. The beads were washed three times, and the supernatant was again replaced with a coupling buffer. Subsequently, the appropriate amount of protein was added to the beads and incubated for 2 h at RT on a rotary shaker at 1000 rpm. After washing the beads, a blocking buffer was added and incubated for 1 h at RT on a rotary shaker at 1000 rpm. Finally, the beads were washed, resuspended in a storage buffer, and stored at 4°C until use.

### 2.8. Conjugated Carboxyl‐Beads Testing Method

Add 1 μL of the SARS‐CoV‐2 N protein and the MERS‐CoV S1 subunit conjugated carboxyl‐beads into one FACS tube together with 50 μL of 1:100 diluted serum sample and incubate for 30 min at RT on a rotary shaker at 600 rpm. After three washes with PBS, 50 μL of the antibody mix was added to the wells, consisting of antihuman IgM‐FITC antibodies (1:100) and antihuman IgG‐PE antibodies (1:200) diluted in PBS, and incubated for 15 min at RT. Next, the tube was washed three times with PBS and acquired with the FACS machine (BD FACSCanto II). The results were analyzed using FlowJo software v10.

### 2.9. Flow Cytometry Parameters and Settings

FACSCanto II was used to acquire all the samples. The test includes two bead populations of APC‐labeled carboxyl beads with different fluorescence intensities to ensure clear separation between SARS‐CoV‐2 conjugated beads and MERS‐CoV beads within the APC channel. Two lasers were used: the 488 nm blue laser for antihuman IgM‐FITC and antihuman IgG‐PE antibodies and the 633 nm red laser for the APC. The samples were acquired at a medium flow rate, with 5000 bead events collected for each sample. A common template was saved for all acquisitions, and compensation was performed with FITC, PE, and APC single‐stained beads. Gating included FSC/SSC bead identification, doublet exclusion, and APC‐based bead discrimination, followed by measurement of FITC (IgM) and PE (IgG) signals. Data were analyzed using FlowJo v10.

### 2.10. Statistical Analysis

The correlation between optical density (OD) values obtained by ELISA and mean fluorescence intensity (MFI) was assessed using linear regression and the associated *R* ^2^ value, and the results were presented as the mean and standard error. We also used Pearson’s correlation analysis to evaluate the relationships between the data obtained from ELISA and the multiplex‐bead immunoassay. The test limit detection was calculated as follows, and regression analysis was performed using GraphPad Prism software: limit of detection (LOD) = 3.3 × standard deviation (SD) of the regression line slope and *y*‐intercept. The sensitivity and specificity binomial 95% CIs of the multiplex‐bead immunoassay were calculated using the Wilson‐Brown method and are shown as percentages with a middle line at the median. *p*‐value style was applied to demonstrate significant differences ( ^∗^ *p* < 0.05,  ^∗∗^ *p* < 0.005,  ^∗∗∗^ *p* < 0.0005,  ^∗∗∗∗^ *p* < 0.0005).

The cut‐off value for each analyte was initially established as the mean MFI (or OD) of the primarily utilized 100 previously confirmed seronegative samples plus three times the SD. To validate these thresholds, receiver operating characteristic (ROC) curve analyses were performed, including both positive and negative samples, and the optimal cut‐off points were selected based on the Youden index (J = sensitivity + specificity−1). For the outlier analysis, negative control measurements for each analyte were evaluated for single‐point anomalies using Grubbs’ test (two‐sided, *α* = 0.05). One extreme value in the negative control group was identified as a statistical outlier (*p*  < 0.05) and excluded prior to the cut‐off calculation. The outliers of the negative control dataset for SARS‐CoV‐2 IgM: *G* = 8.912; SARS‐CoV‐2 IgG: *G* = 7.484; MERS‐CoV IgM: *G* = 11.52; and MERS‐CoV IgG: *G* = 9.581. Intra‐assay precision was assessed by measuring six replicate wells of three samples representing low, medium, and high positive antibody levels for each antigen (SARS‐CoV‐2 N IgM, SARS‐CoV‐2 N IgG, MERS‐CoV S1 IgM, and MERS‐CoV S1 IgG). The coefficient of variation (CV%) was calculated as follows: CV% = SD/Mean × 100. By using the same formula, the Interassay variability was assessed to determine assay reproducibility across separate analytical runs. Low‐, medium‐, and high‐positive serum samples for each antigen were tested on three different days during the study period. Each sample was processed in duplicate in each run. The predefined acceptance threshold for CV is <20% for both intra‐assay and interassay precision, in line with standard analytical validation practices for research‐grade immunoassays.

Linear regression analyses were performed using GraphPad Prism v10. For each dataset, both *R* ^2^ and adjusted *R* ^2^ were calculated. Model assumptions were evaluated using residual plots, including scatter plots of residuals versus fitted values (to assess homoscedasticity and linearity) and residual normality plots. Regression graphs display the best‐fit line with 95% confidence intervals and 95% prediction intervals for individual future observations.

This study used 41 and 58 positive samples and 149 and 132 negative specimens for SARS‐CoV‐2 N protein‐specific IgM and IgG antibodies, respectively. The sample sizes used for the MERS‐CoV S1 subunit were 10 positive and 185 negative samples for IgM and 42 positive and 149 negative samples, unless otherwise stated in the figure legend. Graphical presentations were made using GraphPad Prism version 9.0.2 software (GraphPad Software, Inc., CA, USA) and IBM SPSS software.

## 3. Results

### 3.1. Production of Recombinant MERS‐CoV S1 and SARS‐CoV‐2 N Protein

We have utilized SARS‐CoV‐2 N protein to detect humoral responses in tested individuals. Here, a bacterial expression system was used to express and purify a histidine‐tagged recombinant SARS‐CoV‐2 N protein via i‐NTA affinity chromatography. Supporting Information 1: Figure S1 shows the pure recombinant SARS‐CoV‐2 N protein acquired at the expected size ≈ of 46 kDa, as presented by SDS‐PAGE and western blot analysis using anti‐His tag antibodies. The specificity and antigenicity of the produced protein were previously confirmed, and only seropositive COVID‐19 samples were detected by the produced N protein, not in serum from seronegative subjects [12].

To produce recombinant MERS‐CoV S1, we utilized ExpiCHO‐S cells using PEI‐mediated transfection and transfected with the histidine‐tagged MERS‐CoV S1 plasmid under mild hypothermic conditions. The Ni Sepharose Excel affinity resins that purify histidine‐tagged proteins were used to purify the expressed recombinant protein from the culture supernatant. The expected size of the pure protein, ≈80 kDa, was then confirmed by SDS‐PAGE and western blot analysis (Supporting Information 1: Figure S1).

### 3.2. Detection of MERS‐CoV S1 and SARS‐CoV‐2 N Protein‐Specific IgM and IgG Antibodies by ELISA

**Figure 1:**
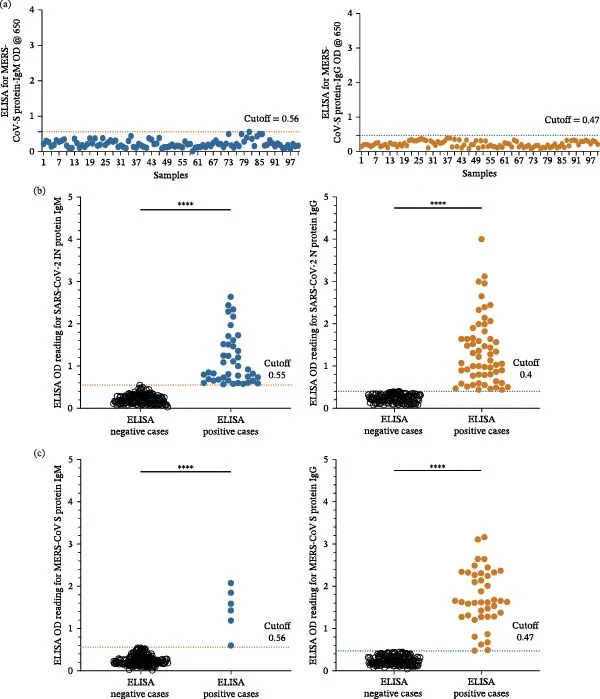
Detection of MERS‐CoV S1 subunit and SARS‐CoV‐2 N protein‐specific IgM and IgG antibodies by ELISA. (a) The 100 negative serum samples were used to calculate the cut‐off values for the in‐house developed ELISA to detect MERS‐CoV S1 subunit IgM and IgG‐specific antibodies. Plots show ELISA OD reading for (b) SARS‐CoV‐2 N protein and (c) MERS‐CoV S1 IgM and IgG for negative and RT‐PCR confirmed cases. Here, 149 negative and 41 positive samples were used for SARS‐CoV‐2 N protein‐specific IgM, while 132 negative and 58 positive samples were used for the IgG antibodies. The 185 negative and 6 positive samples were used for MERS‐CoV N protein‐specific IgM, while 149 negative and 42 positive samples were used for the IgG antibodies. Statistics were calculated by unpaired *t*‐test, and  ^∗^ *p* < 0.05,  ^∗∗^ *p* < 0.005,  ^∗∗∗^ *p* < 0.0005, and  ^∗∗∗∗^ *p* < 0.0005 were considered significant.

To establish the antibody spectrum of the tested serum samples in this study, MERS‐CoV S1‐ and SARS‐CoV‐2 N‐protein‐specific IgM and IgG antibodies were measured using the standard in‐house ELISA method developed and validated in‐house [9, 12]. However, the ELISA previously established for MERS‐CoV S1 was designed to detect IgG‐specific antibodies, not IgM, and was performed using commercially available S1 rather than the in‐house‐generated protein [9]. Therefore, we initially utilized previously confirmed seronegative serum samples collected from healthy individuals to calculate the cut‐off values for MERS‐CoV S1‐specific IgM and IgG antibodies [9]. As shown in Figure 1a, the cut‐off value (mean + 3SD) was found to be 0.56 (mean = 0.226, SD = 0.1109) for MERS‐CoV S1 IgM‐specific antibodies and 0.47 (mean = 0.220, SD = 0.084) for IgG. The mean OD for the ELISA‐negative serum samples for SARS‐CoV‐2 N protein‐specific IgM was 0.2167 (ranging from 0.027 to 0.55) and 0.2346 (ranging from 0.074 to 0.40) for IgG antibodies (Figure 1b). As for the negative serum samples that were used for the MERS‐CoV S1 IgM and IgG‐specific antibodies, the ELISA OD mean values were 0.2424 (ranging from 0.0137 to 0.540) for the IgM and 0.2417 (ranging from 0.0423 to 0.444) for the IgG‐specific antibodies (Figure 1c). As expected, the mean OD values for the positive serum samples used in this study were significantly higher than those of the negative specimens. As shown in Figure 1b, the OD mean for SARS‐CoV‐2‐specific IgM was 1.150 (ranging from 0.57 to 2.640) and 1.357 (ranging from 0.44 to 4) for IgG‐specific antibodies. The mean OD for the MERS‐CoV S1‐specific IgM was reported here at a value of 1.457 (ranging from 0.6 to 2.08) and 1.738 (ranging from 0.480 to 3.167) for the IgG‐specific antibodies (Figure 1c).

### 3.3. Development of the In‐House Multiplex‐Bead Immunoassay to Detect Humoral Responses for MERS‐CoV S1 and SARS‐CoV‐2 N Protein Simultaneously

**Figure 2:**
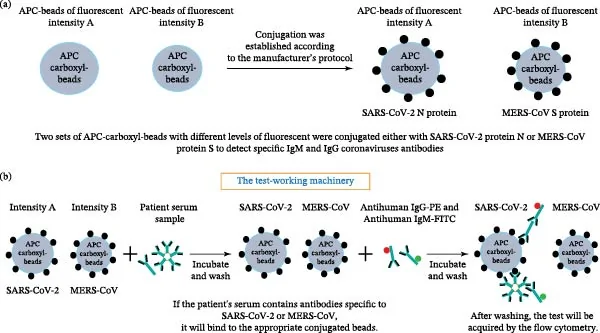
The in‐house multiplex‐bead immunoassay components and principle. (a) Schematic of the components of the developed multiplex‐bead immunoassay. (b) Illustration of the principle used in our developed multiplex‐bead immunoassay: samples were diluted (1:100) and incubated with the multiplex‐bead for 30 min at RT, then stained with antihuman IgM‐FITC (1:100) and antihuman IgG‐PE (1:200) for 15 min at RT.

**Figure 3:**
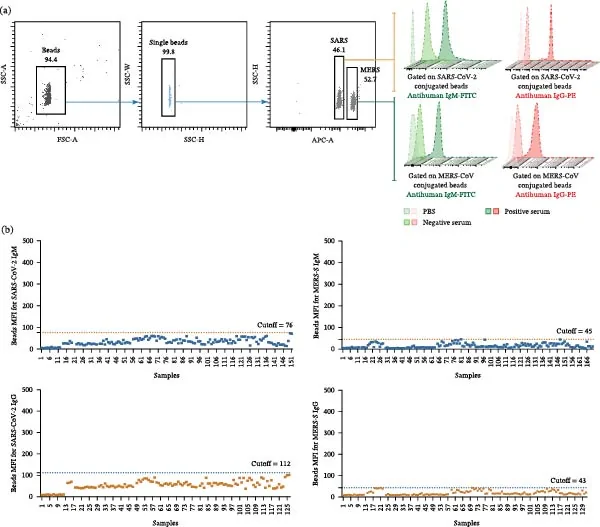
Development of the in‐house multiplex‐bead immunoassay to detect humoral responses for SARS‐CoV‐2 N protein and MERS‐CoV S1 subunit simultaneously. (a) Gating strategies used by the FlowJo software for the results obtained using the multiplex‐bead immunoassay. Samples were diluted (1:100) and incubated with the multiplex‐bead for 30 min at RT, then stained with antihuman IgM‐FITC (1:100) and antihuman IgG‐PE (1:200) for 15 min at RT before acquisition. (b) Cut‐off values for the developed multiplex‐bead immunoassay, which was determined by using seronegative serum samples for SARS‐CoV‐2 N protein IgM (*n* = 151), IgG (*n* = 126), and for MERS‐CoV S1 IgM (*n* = 170) and IgG (*n* = 131). The cut‐off values were calculated as the mean + 3 SD.

Here, we developed a platform to detect humoral responses (IgM and IgG) to more than one targeted pathogen in a single run using a single sample. In Figure 2a, two carboxyl beads of APC with different fluorescent intensities were conjugated. One bead was conjugated with the SARS‐CoV‐2 N protein, and the second was labeled with MERS‐CoV S1 at a 1 μg concentration per 10 million beads. To detect the specific humoral responses to SARS‐CoV‐2 N protein and MERS‐CoV S1, after several steps of optimization to achieve a functional assay with a short overall turnaround time, the test machinery consisted of the following steps. The patient serum at 1:100 was added to only 1 μL of the conjugated mix of beads. After incubation and washing, antihuman IgM‐FITC and antihuman IgG‐PE antibodies were added to the reaction. Upon the presence of IgM, IgG, or both in the sample, these antibodies bind specifically to the appropriate beads, and the results are acquired on the FACS machine (Figure 2b). Figure 3a shows the gating strategies used to analyze and obtain the final results. Notably, this platform can be extended to detect more specific antibodies against additional pathogens by using carboxyl‐beads of APC with different fluorescent intensities than those included in this assay.

Next, we used serum samples from healthy individuals who tested negative on ELISA to calculate the test cut‐off values (mean + 3 SD). As shown in Figure 3b, the cut‐off values for SARS‐CoV‐2 N protein were 76 (mean = 31, SD = 15) for IgM and 112 (mean = 49.9, SD = 20.7) for IgG‐specific antibodies. As for MERS‐CoV S1, the cut‐off values were 45 (mean = 15, SD = 10) for IgM and 43 (mean = 14.2, SD = 9.6) for IgG‐specific antibodies (Figure 3b). No false‐positive results were observed in the samples used to calculate the assay’s cut‐off values, except for one negative control sample that showed an extremely high MFI value across all targets in the test, which was excluded based on Grubbs’ outlier testing (two‐sided, α = 0.05). Moreover, ROC analyses confirmed excellent assay performance, with area under the curve (AUC) values ranging from 0.927 to 0.992 across all analytes. The ROC‐derived optimal cut‐off values were largely consistent, with slight differences relative to those calculated by the mean + 3SD approach, supporting the robustness of our original threshold determination (Supporting Information 2: Figure S2). The slight difference in the cut‐off values obtained from the two methods reflects their different purposes. Calculating the cut‐off values using mean + 3SD of the negative control shows the minimal detectable value above the background. In contrast, the ROC cutoff aims to optimize diagnostic discrimination using Youden’s index. Both methods were used in this study to ensure transparency and assay reproducibility.

### 3.4. Determination of the Test LOD and Correlation With ELISA

**Figure 4:**
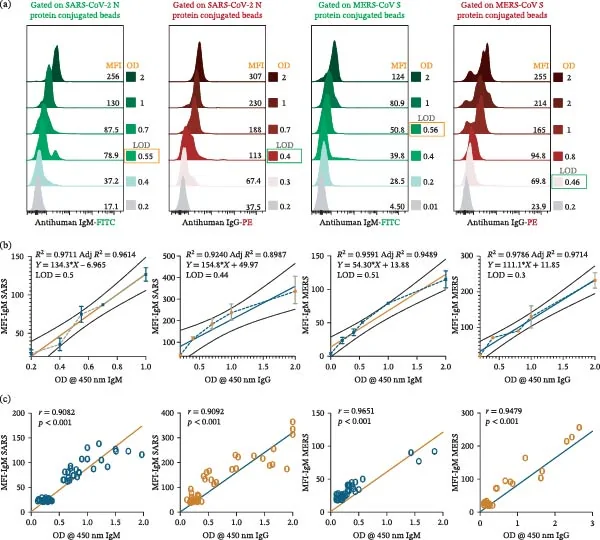
Determination of the test limit of detection and correlation with ELISA. Serum samples of known OD values ranging from 0.2 to 2 were utilized with the developed multiplex‐bead immunoassay and analyzed with FlowJo software. (a) FACS‐histograms show the difference in the MFI values determining the LOD for each target in the assay. (b) The linear relationship between known ELISA OD reading for the SARS‐CoV‐2 N protein and MERS‐CoV S1‐specific IgM and IgG antibodies, and the MFI values obtained via FlowJo analysis, each experiment was done three independent times, with one test per experiment. (c) Pearson’s correlation was used to evaluate the relationships between the data obtained from ELISA and the multiplex‐bead immunoassay for SARS‐CoV‐2 N protein IgM (*n* = 46) and IgG (*n* = 40), and the MERS‐CoV S1 subunit IgM (*n* = 36) and IgG (*n* = 30).

Having established the serum sample profile, specimens with known OD values obtained from SARS‐CoV‐2 N protein and MERS‐CoV S1 ELISAs, which detect those pathogens’ specific humoral responses, IgM and IgG, were used in triplicate to determine the assay LOD. However, the MERS‐CoV S1‐specific IgM antibody samples were limited to a serum with an OD reading of 0.57 (only one sample) and to serum samples with an OD value of 2 (only two samples). Thus, the test was performed only once and twice with the multiplex‐bead immunoassay spontaneously. Based on the definition of LOD, the lowest detectable amount of an analyte, the LOD for the assay has reliably been detected at a cut‐off similar to the levels of the OD values of the ELISA for all analytes (Figure 4a) [2, 3, 13, 14]. As shown in Figure 4a, the LOD values of the SARS‐CoV‐2 N protein assay were 78.9 (at OD reading of 0.55) for IgM and 113 (at OD reading of 0.4) for IgG, whereas for MERS‐CoV S1, the LOD values were 50.8 (at OD reading of 0.57) for IgM and 69.8 (at OD reading of 0.46) for IgG antibodies. The data shown in Figure 4b confirm the LOD obtained, which was calculated from the calibration curve by plotting the assay MFI values against the ELISA OD readings. Figure 4b shows that the developed multiplex‐bead immunoassay for SARS‐CoV‐2 N protein and MERS‐CoV S1, IgM, and IgG‐specific antibodies has provided a good linear dynamic. The latter ranged from plotting MFI values versus ELISA OD readings from 0.5 (SARS‐CoV‐2 IgM) and 0.4 (SARS‐CoV‐2 IgG) to 2 OD, as for MERS‐CoV, ranging from 0.51 (IgM) and 0.3 (IgG) to 2 OD. Therefore, the developed multiplex‐bead immunoassay can detect more than one analyte in a single sample and has LODs identical to those of the standard test used to detect humoral immune responses in serum.

Moreover, the calculations included the adjusted *R* ^2^ values, prediction intervals, and residual diagnostics. For each analyte, adjusted *R* ^2^ values remained consistent with the *R* ^2^ values, confirming good model fit (Figure 4b). SARS‐CoV‐2 IgM demonstrated a strong correlation with ELISA values (slope = 134.3, *R* ^2^ = 0.9711, adjusted *R* ^2^ = 0.9614, and Sy.*x* = 8.12), as did SARS‐CoV‐2 IgG (slope = 154.8, *R* ^2^ = 0.9240, adjusted *R* ^2^ = 0.8987, and Sy.*x* = 36.18). Similarly, high agreement was observed for MERS‐CoV IgM (slope = 54.30, *R* ^2^ = 0.9591, adjusted *R* ^2^ = 0.9489, and Sy.*x* = 9.09) and MERS‐CoV IgG (slope = 111.1, *R* ^2^ = 0.9786, adjusted *R* ^2^ = 0.9714, and Sy.*x* = 13.32). Residual plots demonstrated no major deviations from linearity, no evidence of heteroscedasticity, and approximately normally distributed residuals (Supporting Information 3: Figure S3). In addition, 95% prediction intervals have also been added to each regression plot (Supporting Information 3: Figure S3) to reflect uncertainty in individual measurement prediction rather than the mean trend alone. Prediction envelopes were visibly broader for MERS‐CoV IgM and IgG, consistent with the smaller number of paired data points and reduced signal dispersion compared with SARS‐CoV‐2.

Additionally, the data from the developed multiplex‐bead immunoassay were correlated with the ELISA OD readings using Pearson’s correlation analysis to assess variability between the two assays. The analysis shown in Figure 4c indicated that the ELISA OD readings and MFI values were positively correlated across all four analytes included in the developed assay. As seen in Figure 4c, the *R*‐value ranged from 0.9082 to 0.9651, with a *p*‐value < 0.001. Moreover, the linear regression for SARS‐CoV‐2 IgM showed *F*_(1,30)_ value of 230.6 (*p* < 0.0001; *Y* = 73.49 × *X* + 1), for IgG the *F*_(1,39)_ value was 462.6 (*p* < 0.0001; *Y* = 159.9 × *X* + 1), as for the MERS‐CoV IgM *F*_(1,35)_ value of 265 (*p* < 0.0001; *Y* = 60.08 × *X* + 1), for IgG the *F*_(1,29)_ value was 431.7 (*p* < 0.0001; *Y* = 81.29 × *X* + 1). These data indicate that the developed multiplex assay provides higher values than ELISA.

### 3.5. The Validation of the Developed Multiplex‐Bead Immunoassay’s Sensitivity, Specificity, and Precision

**Figure 5:**
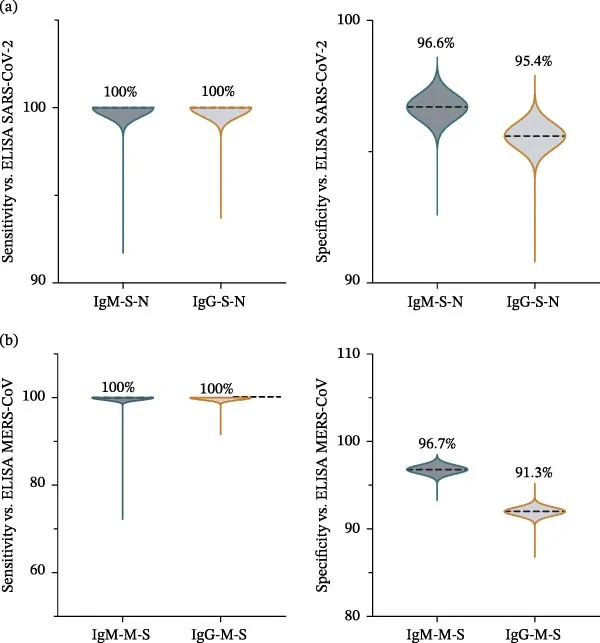
The validation of the developed multiplex‐bead immunoassay’s sensitivity and specificity. Floating plots show the sensitivity and specificity of the in‐house developed multiplex‐bead immunoassay for (a) SARS‐CoV‐2 N protein and (b) MERS‐CoV S1 humoral responses compared to ELISA, the percentages at the top of each plot, and the 95% confidence intervals of IgM and IgG antibodies. Statistics were calculated using the Wilson/Brown methods.

After confirming that the developed multiplex‐bead immunoassay can detect coronavirus‐specific humoral responses in patient serum with good correlation with ELISA, the standard method, we evaluated the assay’s sensitivity and specificity. Although ELISA and the developed multiplex‐bead immunoassay provide quantitative readouts, results were considered positive when exceeding the calculated cut‐off value. Figure 5a,b shows that the sensitivity of the developed multiplex‐bead immunoassay for SARS‐CoV‐2 and MERS‐CoV IgM and IgG was 100% compared with ELISA. Here, the multiplex‐bead immunoassay detecting SARS‐CoV‐2 IgM had 100% sensitivity (95% CI: 91.8%–100%; 43/43). Also, the assay has shown 100% sensitivity for detecting SARS‐CoV‐2 IgG antibodies (95% CI: 93.8%–100%; 58/58). As for the MERS‐CoV multiplexing‐bead immunoassay, upon detecting MERS‐CoV IgM antibodies, the assay showed 100% sensitivity (95% CI: 72.2%–100%; 10/10), and the sensitivity of IgG was also 100% (95% CI: 91.6%–100%; 42/42). The assay’s specificity against ELISA has also been high; here, we have included more samples than the one used to calculate the assay cut‐off value, and we have obtained several false‐positive results. As shown in Figure 5a,b, the developed assay specificity upon detecting SARS‐CoV‐2 IgM was 96.6% (95% CI: 92.7–98.6%; 150/155) and 95.4% for the IgG antibodies (95% CI: 90.8%–98%; 132/138). Hence, these results demonstrate that the developed multiplex‐bead immunoassay is highly sensitive and specific for the target analyte compared with the standard method for detecting humoral responses in serum.

The multiplex‐bead immunoassay demonstrated substantial intra‐assay precision across all analytes (Table 1). For SARS‐CoV‐2 N IgM, CV% values were 7.23% (low), 0.88% (medium), and 2.38% (high). SARS‐CoV‐2 N IgG exhibited CV values of 2.72%, 1.25%, and 0.14%, respectively. For MERS‐CoV S1, IgM CV values were 4.40%, 3.32%, and 2.01%, while IgG CVs were 6.81%, 1.77%, and 1.94%. All values were well below the prespecified acceptance threshold (<15%–20%), confirming excellent repeatability and analytical stability of the assay.

**Table 1:** Intra‐assay precision of the in‐house developed multiplex‐bead assay.

Analyte	Low positive (CV%)	Medium positive (CV%)	High positive (CV%)
SARS‐CoV‐2 IgM	7.23	0.88	2.38
SARS‐CoV‐2 IgG	2.72	1.25	0.14
MERS‐CoV IgM	4.40	3.32	2.01
MERS‐CoV IgG	6.81	1.77	1.94

To evaluate the assay’s reproducibility across independent runs, interprecision was measured using low‐, medium‐, and high‐positive serum samples tested on three different days. The CV% values for SARS‐CoV‐2 IgM ranged from 1.41% to 4.49% for low samples, 0.45–1.43% for medium samples, and 0.27–1.10% for high samples. For SARS‐CoV‐2 IgG, CV% ranged from 1.79% to 3.07% for low samples, 0.74–1.99% for medium samples, and 0.07–0.38% for high samples. CV% values for MERS‐COVID‐19 IgM were 2.28–8.16%, 4.49–6.63%, and 2.27–3.78% for low, medium, and high samples, respectively. MERS‐COVID‐19 IgG CV% ranged from 2.03 to 6.63% for low samples, 1.48–2.58% for medium samples, and 0.54–1.37% for high samples. All interassay CV% values were below 20%, confirming the high reproducibility of the developed multiplex immunoassay (Table 2). Table 3 summarizes the assay performance results.

**Table 2:** Interassay precision of the in‐house developed multiplex‐bead assay.

Analyte	Low positive (CV%)	Medium positive (CV%)	High positive (CV%)
SARS‐CoV‐2 IgM	4.49, 2.49, 1.41	1.20, 0.45, 1.43	1.10, 0.27, 0.53
SARS‐CoV‐2 IgG	1.79, 3.01, 3.07	1.99, 0.76, 0.74	0.38, 0.07, 0.22
MERS‐CoV IgM	8.16, 2.28, 8.05	4.49, 6.63, 4.49	2.27, 3.66, 3.78
MERS‐CoV IgG	2.03, 6.43, 3.29	1.48, 2.58, 2.27	1.37, 0.54, 1.06

**Table 3:** A summary of the assay performance results.

Assay	Sensitivity (%)	Specificity (%)	LOD	*R* ^2^
SARS‐CoV‐2 IgM	100	96.6	75.55	0.9711
SARS‐CoV‐2 IgG	100	95.4	94.4	0.9240
MERS‐CoV IgM	100	96.7	49.5	0.9591
MERS‐CoV IgG	100	91.3	45.5	0.9786

### 3.6. Advantages of the Developed Multiplex‐Bead Immunoassay Over ELISA

Unlike ELISA, which can detect only one analyte per test within at least 4 h, the developed multiplex‐bead immunoassay can detect multiple analytes within 1 h and 20 min. Therefore, this method has overcome the high time‐over and limited data associated with ELISA. The high data provided by the multiplex‐bead immunoassays reflect on the cost of the assay, making it more attractive and cheaper than ELISA. Table 4 shows a detailed comparison between the in‐house‐developed ELISA and the in‐house‐developed multiplex‐bead immunoassay. The table shows the significant difference in cost and time between the two assays, with the multiplex‐bead immunoassay having an overall cost of $0.8162 and the ELISA having an overall cost of $6.2264 for detecting all analytes. Also, the table shows the times for the two assays, with the overall completion time of the multiplex‐bead immunoassay at about 1.2 h. In contrast, the overall ELISA completion time for the one analyte detectable is about 5 h. Thus, the developed multiplex‐bead immunoassay is superior to the ELISA in terms of cost, time, and the amount of data obtained.

**Table 4:** Comparison between the in‐house developed multiplex‐beads assay and ELISA.

Items	Multiplex cost	ELISA cost	Company (USA)
SARS‐CoV‐2 N protein	1 μg/mL = $3.2 10 million coated beads in 1 mL = $3.2 10 k beads used/test = $0.0032	1 μg/mL = $3.2 4 μg/mL = $12.8 Per plate (10 mL) = $128 4 μg/mL per well = $1.33	In‐house
MERS‐CoV S protein	1 μg/mL = $20 10 million coated beads in 1 mL = $20 10 k beads used/test = $0.02	1 μg/mL = $20 2 μg/mL = $40 Per plate (10 mL) = $400 2 μg/mL per well = $4.16	In‐house
Plates (96 well)	N/A	Flat‐bottom/50 case = $255 1 plate = $5.1 1 well = $0.053	Thermofisher, Cat: 3455; Cat: 277143
Tubes	1000 tubes = $140 1 tube = $0.14	N/A	Fisherscientific, Cat: 14‐959‐5
PBS	500 mL PBS = $29.1 1.3 mL/test = $0.075	500 mL PBS = $29.1 0.1 mL/well = $0.0058	Fisherscientific, Cat: 10010023
PBS‐Tween‐20 (0.05%)	N/A	500 mL PBS‐*T* = $29.28 2.4 mL/well = $0.14	Sigmaaldrich; Cat: p1379‐25ml
Blocking buffer (PBS‐T + 5% skim milk)	N/A	500 mL = $31.99 0.5 mL/well = $0.032	Sigmaaldrich, Cat:1153630500
Stop solution	N/A	500 mL stop solution = $8.62 0.1 mL/well = $0.0017	Fisherscientific, Cat: A300‐500
Beads	1000 μL = $177 1 μL = $0.17	N/A	Biolegend, Cat: 740168
Conjugated antibodies	Antihuman IgM‐FITC 0.5 mL = $188 0.5 μL /test = $0.188 Antihuman IgG‐PE 0.5 mL = $323 0.25 mL/test = $0.16	HRP‐antihuman IgG 1.5 mL = $134 0.05 μL/well = $0.0044 HRP, antihuman IgM 1.5 mL = $161 0.05 μL/well = $0.0053	Biolegend, Cat: 314506; Cat: 410708 Jackson ImmunoResearch, Cat: 109‐035‐088; Cat: 109‐035‐129
Substrate	N/A	100 mL = $126 0.1 mL/well = $0.126	Sigmaaldrich; Cat: T4444‐100ml
FACS sheets (BD FACSCanto II)	Sheets/20 L = $73 1 h uses 1 L = $3.65 1 min/test = $0.06	N/A	Fisherscientific, Cat: NC0022088
Total cost	One test detects both SARS‐CoV‐2 and MERS‐CoV, simultaneously. Therefore, the test cost: $0.8162	ELISA detect analytes separately. Therefore, the test cost for both analyte is: $6.2264	—
Preparation time	1.2 h including washing and aquation	About 5 h including washing and OD reading for each analyte	—

*Note:* Cost analysis reflects only the prices of reagents and consumables. Flow cytometer investment (typically USD $80,000–$150,000) is not included in the implementation cost and should be considered when evaluating implementation costs, although such instruments are increasingly available in clinical laboratory settings.

## 4. Discussion

Despite widespread vaccination against SARS‐CoV‐2, MERS‐CoV continues to spread endemically in some regions where no vaccine exists. Although RT‐PCR was the primary detection method during the COVID‐19 pandemic, it has several limitations, including high costs, long turnaround times, and drawbacks of rapid antigen testing, particularly false negatives, that emphasize the ongoing importance of serological assays. These assays can indicate the stage of infection by detecting humoral immune responses [4–7]. Serology also provides valuable insights into immune responses after vaccination, potential associations with disease severity or outcomes, and unique epidemiological data such as estimates of undetected infections in the community [15–20]. As a result, multiple reports recommend combining these methods to improve accuracy and control disease spread [8, 21, 22]. Consequently, several serological assays, both commercially available and developed in‐house, have been created to assist clinical diagnosis, improve assay specificity and sensitivity, and standardize results across different techniques [23–25].

Respiratory viral infections share similar symptoms, and relying solely on symptoms, imaging, and viral detection tests for diagnosis is insufficient, especially since the viral load in the upper respiratory tract decreases over time [26–28]. Viruses differ in their progression and treatment; thus, an accurate diagnosis is vital. These factors also support including serology in the testing panel to identify the infectious agent and determine the disease stage. Therefore, developing a cost‐effective and rapid platform to detect humoral responses is crucial as it enables accurate diagnosis and ongoing surveillance, particularly among asymptomatic individuals, to prevent disease transmission [8, 10, 29–31].

In this study, we successfully developed an in‐house multiplex‐bead immunoassay platform to detect specific humoral responses against multiple coronaviruses, with high specificity and sensitivity ranging from 95% to 100%. Compared to the standard method used to detect humoral responses in patients’ serum samples, this platform offers many cost, time, sensitivity, and specificity advantages. While numerous publications have documented various bead‐based immunological assays, the work presented here still makes a valuable contribution to the scientific literature. Most, if not all, of the previously published works have used commercially available proteins to coat the microbeads utilized in their developed assays [15, 16, 21, 22]. Moreover, studies that have established flow cytometry‐based bead assays for serological detection of SARS‐CoV‐2 have detected only SARS‐CoV‐2 humoral responses by including several SARS‐CoV‐2 antigens or by detecting multiple SARS‐CoV‐2 variants. Limited studies have examined MERS‐CoV humoral responses, and these studies have only developed assays to detect IgG using the close system Luminex [17, 18]. Unlike others, we have provided a detailed cost estimate for assay development using in‐house materials, except for the commercially purchased beads, and after optimizing the assay to obtain a cost‐effective test without jeopardizing its sensitivity or specificity. Like other platforms, the one provided here can be expanded to include more targets within the same cost range.

Several variants of SARS‐CoV‐2 have emerged globally. Most variations, however, have occurred within the spike protein, with few mutations in the N protein; thus, the N protein gene is more stable and conserved than the S protein gene [19, 20]. Although this may raise concerns regarding many existing SARS‐CoV‐2 therapies and vaccines, we have utilized the SARS‐CoV‐2 N protein to detect humoral responses produced by infection with any variant. The SARS‐CoV‐2 N protein has been employed for routine nucleic acid and serological testing for several reasons, including its high frequency and presence in infected individuals but not in vaccinated subjects [23, 24]. Therefore, using the SARS‐CoV‐2 N protein enables us to distinguish between naturally infected and vaccinated patients as the N protein is absent in all currently vaccinated individuals. We recognize that antibodies against the RBD correlate more strongly with neutralization activity [25]; hence, our antigen choice reflects a trade‐off that prioritizes infection/vaccination differentiation over neutralization correlation. Future iterations of the assay platform will include both N and RBD to provide a more comprehensive serological profile. Given that cross‐reactivity between the SARS‐CoV‐2 and MERS‐CoV S proteins is known to be limited [32, 33]. Moreover, the S2 subunit is more conserved across coronaviruses than S1, and S1 is more variable between CoVs [26–29]. Consequently, the MERS‐CoV S1 subunit has been selected to minimize cross‐reactivity between the two beads. Moreover, unlike the SARS‐CoV‐2 S protein, which has experienced several significant mutations leading to major variants, the MERS‐CoV S protein has shown limited mutations [30, 31, 34]. Furthermore, specific antibodies against the MERS‐CoV S protein will be detected in infected individuals, particularly in the absence of MERS‐CoV vaccines.

Our results demonstrate that the two selected proteins provide clear signals with high sensitivity and specificity compared to ELISA. In agreement with several reports documenting the lack of cross‐reactivity between the SARS‐CoV‐2 S1 subunit and other coronaviruses, we have likewise reported no cross‐reactivity between antibodies produced against the MERS‐CoV S1 subunit and SARS‐CoV‐2, achieving approximately a 100% sensitivity rate with both developed beads [35, 36].

Our study has limitations, primarily because we have created a platform to detect humoral responses against only two targets. However, by applying the same testing principles and following the same development steps, this platform has the potential to be expanded to include additional analytes. Although the test design allows for the theoretical expansion of additional targets, this study validated only two analytes. Therefore, the platform has been described as having potential for expansion, recognizing that practical scalability must be established in future work. Moreover, MERS‐CoV IgM validation included only 10 confirmed‐positive samples, resulting in 100% sensitivity (95% CI: 72.2%–100%). The small sample size resulted in wide confidence intervals, limiting the precision of these estimates. Therefore, the MERS‐CoV results here are interpreted as preliminary proof‐of‐concept data illustrating the multiplexing capability of the developed assay. Expanded testing with larger sample sets is ongoing to further validate the performance for MERS‐CoV and other respiratory pathogens. Notably, although the present study did not include direct cross‐reactivity testing with endemic human coronaviruses (HCoV‐OC43 and HCoV‐229E), the high specificity observed using prepandemic sera suggests limited cross‐reactivity. These negative control samples, collected before 2019, were expected to contain antibodies to circulating seasonal HCoVs; yet, no significant false‐positive reactivity was detected against SARS‐CoV‐2 or MERS‐CoV antigens. Furthermore, the use of recombinant SARS‐CoV‐2 N protein and MERS‐CoV S1 subunit, which share low sequence similarity in antigenic regions, minimizes the risk of serological cross‐reactivity. Nevertheless, future work will include systematic testing with well‐characterized HCoV‐positive sera and SARS‐CoV‐2‐positive samples to further confirm the assay specificity across multiple coronaviruses.

## 5. Conclusion

Given the importance of a cost‐effective, time‐efficient method for detecting humoral responses to respiratory viral infections with high specificity and sensitivity, after significant optimization, we have developed a test that uses minimal reagents and can be performed quickly to detect multiple analytes simultaneously. The developed multiplex‐bead immunoassay correlates well and has detection limits comparable to those of the standard ELISA method.

## Author Contributions

Rahaf Y. Alhabbab conceptualized the study, designed and performed experiments, analyzed data, wrote the manuscript. Mohammad Abdullah Basabrain, Abdulaziz Kulthum, Sawsan Alamri, Maimonah Alghanmi, Asem Alsharef, and Tarfa A. Altorki performed some experiments, revised the manuscript, analyzed some data. Rahaf Y. Alhabbab supervised the project. Anwar M. Hashem cosupervised the project. Wesam H. Abdulaal revised the manuscript.

## Funding

This study was funded by Community Jameel in Saudi Arabia under the Jameel Fund for Infectious Disease Research and Innovation (Grant JML:007‐141‐2024).

## Ethics Statement

This study was performed based on ethical approvals from the Institutional Review Board at the Ministry of Health, Saudi Arabia (IRB Number H‐02‐J‐002 and Project Number 1367).

## Conflicts of Interest

The authors declare no conflicts of interest.

## Supporting Information

Additional supporting information can be found online in the Supporting Information section.

## Supporting information


**Supporting Information 1** Figure S1. Production of recombinant SARS‐CoV‐2 N protein and MERS‐CoVCOVID‐19 S1 subunit. Recombinant SARS‐CoV‐2 N protein and MERS‐CoVCOVID‐19 S1 protein were identified with SDS‐PAGE and western blot using anti‐His tag antibodies. The SDS‐PAGE and the western‐blot of the SARS‐CoV‐2 N protein showed the protein at ≈46 kDa, while the MERS‐CoVCOVID‐19 S1 protein showed the protein at ≈80 kDa.

**Supporting Information 2** Figure S2. ROC curve analysis to determine the cut‐off values. The ROC analysis was performed using both positive and negative samples, and the optimal cut‐off was determined using Youden’s index, as indicated, with corresponding sensitivity and specificity values listed beside the plot. ROC curve and data are shown for (a) SARS‐CoV‐2 IgM (positive *n* = 41, negative *n* = 150), (b) SARS‐CoV‐2 IgG (positive *n* = 58, negative *n* = 121), (c) MERS‐CoVCOVID‐19 IgM (positive n = 10, negative n = 161), (d) MERS‐CoVCOVID‐19 IgG (positive *n* = 42, negative *n* = 126).

**Supporting Information 3** Figure S3. Residual plots to assess model assumptions and linear regression with 95% prediction intervals. (a) residual plots for SARS‐CoV‐2 IgM, SARS‐CoV‐2 IgG, MERS‐CoVCOVID‐19 IgM, and MERS‐CoVCOVID‐19 IgG comparing residual values across the range of ELISA input measurements. In all four models, residuals were symmetrically distributed around zero with no visible curvature or heteroscedasticity, supporting the appropriateness of linear regression. (b) linear regression with 95% prediction intervals, demonstrating the goodness of fit for the final regression models. Points near the line of identity indicate strong predictive agreement between multiplex‐bead immunoassay fluorescence intensity and corresponding ELISA optical density measurements. Each experiment was done three independent times with one test per experiment.

## Data Availability

The data supporting this study’s findings are available upon request from the corresponding author.
