# Supplementary material for: Cost‐Effective and Time‐Saving Multiplex‐Beads Immunoassay Method to Detect Humoral Immune Responses Against Respiratory Infections
Source: J Immunol Res. 2026 Aug 26;2026:8000849. doi: 10.1155/jimr/8000849 (PMC13507927; doi:10.1155/jimr/8000849)
Supplement: Supplementary file 1 [file JIMR-2026-8000849-s001.pdf]

a)

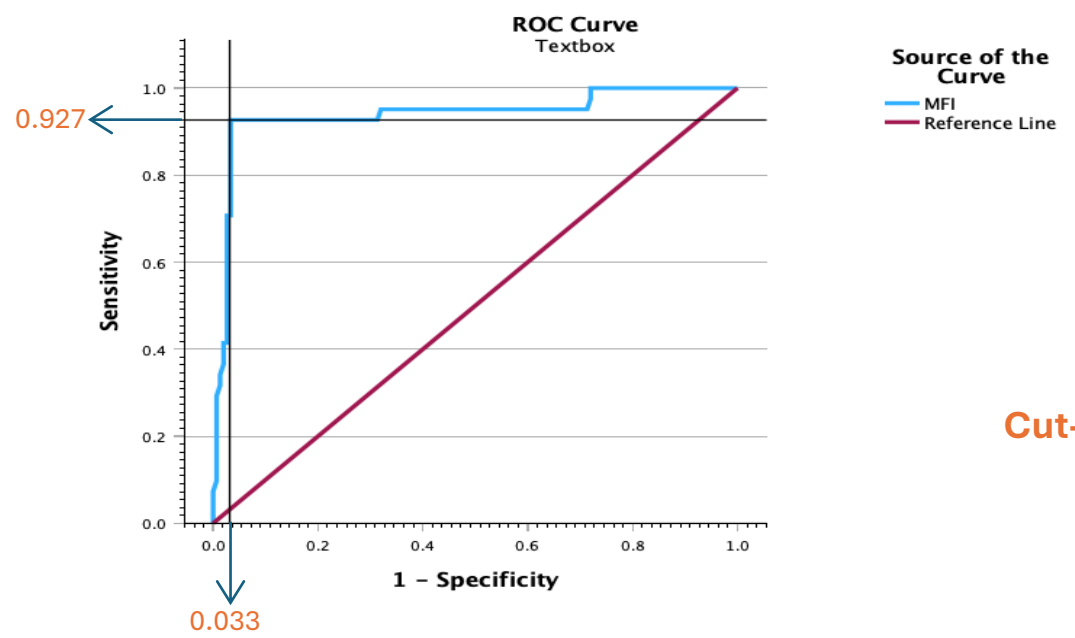

SARS-CoV-2 IgM

Coordinates of the ROC Curve

Test Result Variable(s): MFI

	Positive if Greater Than or Equal To[a]	Sensitivity	1 - Specificity
	70.2500	.927	.047
	71.8000	.927	.040
Cut-off	75.5500	.927	.033
	78.6000	.878	.033
	79.6000	.854	.033

b)

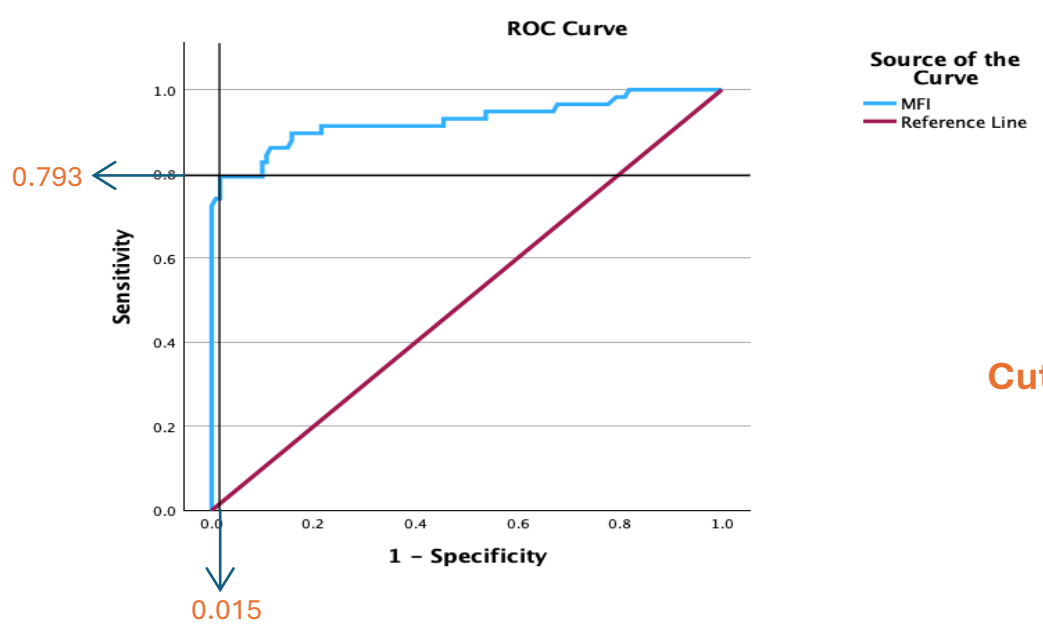

SARS-CoV-2 IgG

Coordinates of the ROC Curve

Test Result Variable(s): MFI

	Positive if Greater Than or Equal To[a]	Sensitivity	1 - Specificity
	89.8000	.793	.033
	91.3500	.793	.025
Cut-off	94.4000	.793	.017
	96.5500	.776	.017
	97.8000	.759	.017

c)

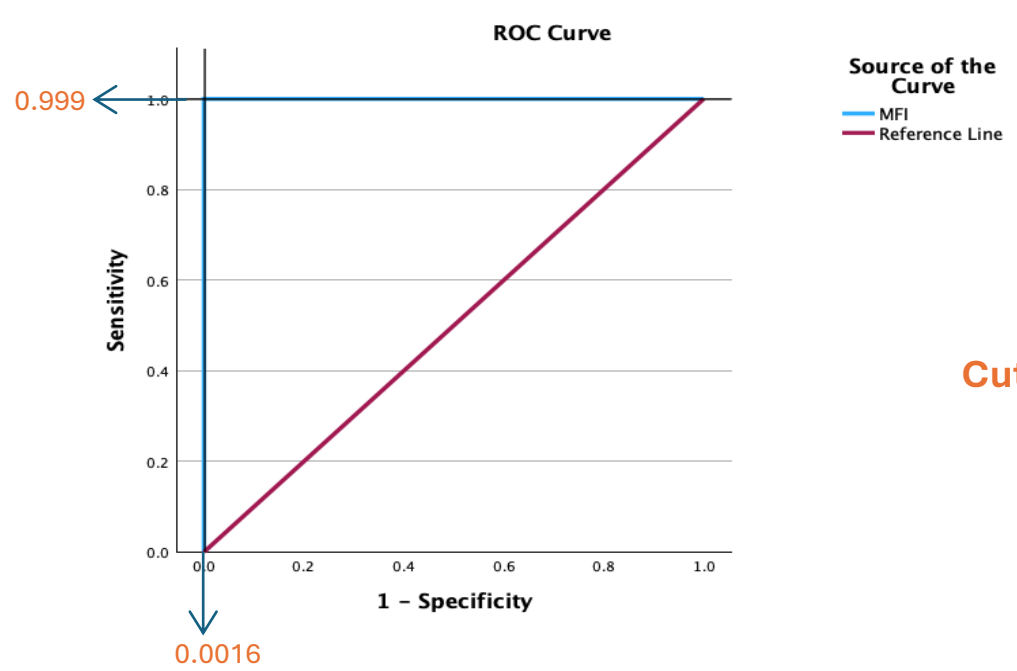

MERS-CoV IgM

Coordinates of the ROC Curve

Test Result Variable(s): MFI

	Positive if Greater Than or Equal To[a]	Sensitivity	1 - Specificity
	38.3500	1.000	.006
	44.4000	1.000	.000
Cut-off	49.5000	.900	.000
	52.5000	.700	.000
	61.0000	.500	.000

d)

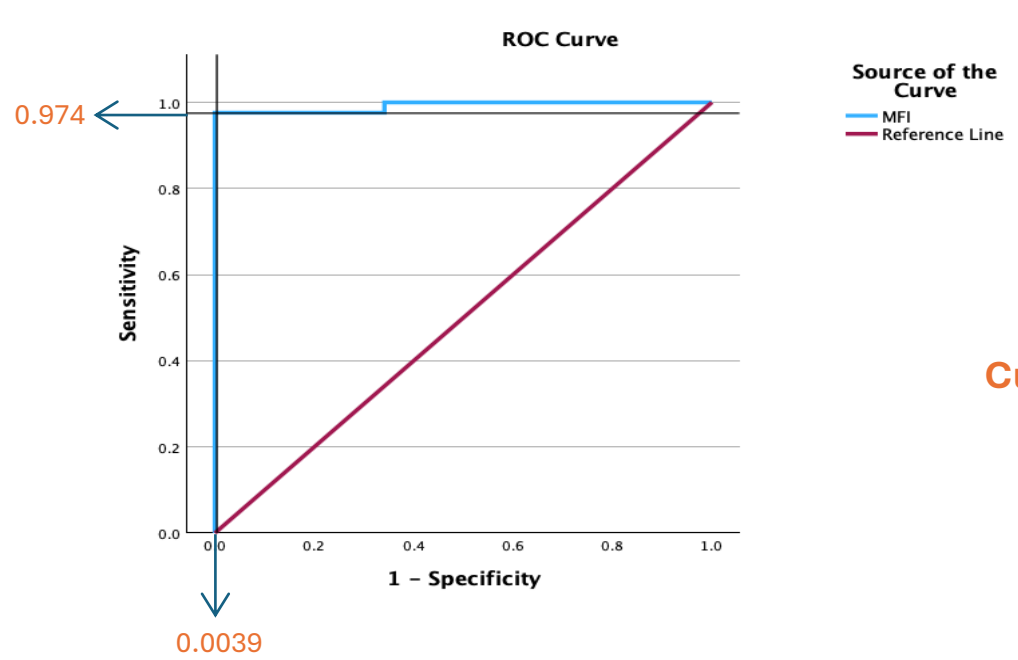

MERS-CoV IgG

Coordinates of the ROC Curve

Test Result Variable(s): MFI

	Positive if Greater Than or Equal To[a]	Sensitivity	1 - Specificity
	42.1500	.976	.008
	44.1500	.976	.000
Cut-off	45.5000	.952	.000
	48.6500	.929	.000
	52.1000	.905	.000
