# Supplementary material for: Cost‐Effective and Time‐Saving Multiplex‐Beads Immunoassay Method to Detect Humoral Immune Responses Against Respiratory Infections
Source: J Immunol Res. 2026 Aug 26;2026:8000849. doi: 10.1155/jimr/8000849 (PMC13507927; doi:10.1155/jimr/8000849)
Supplement: Supplementary file 2 [file JIMR-2026-8000849-s002.pdf]

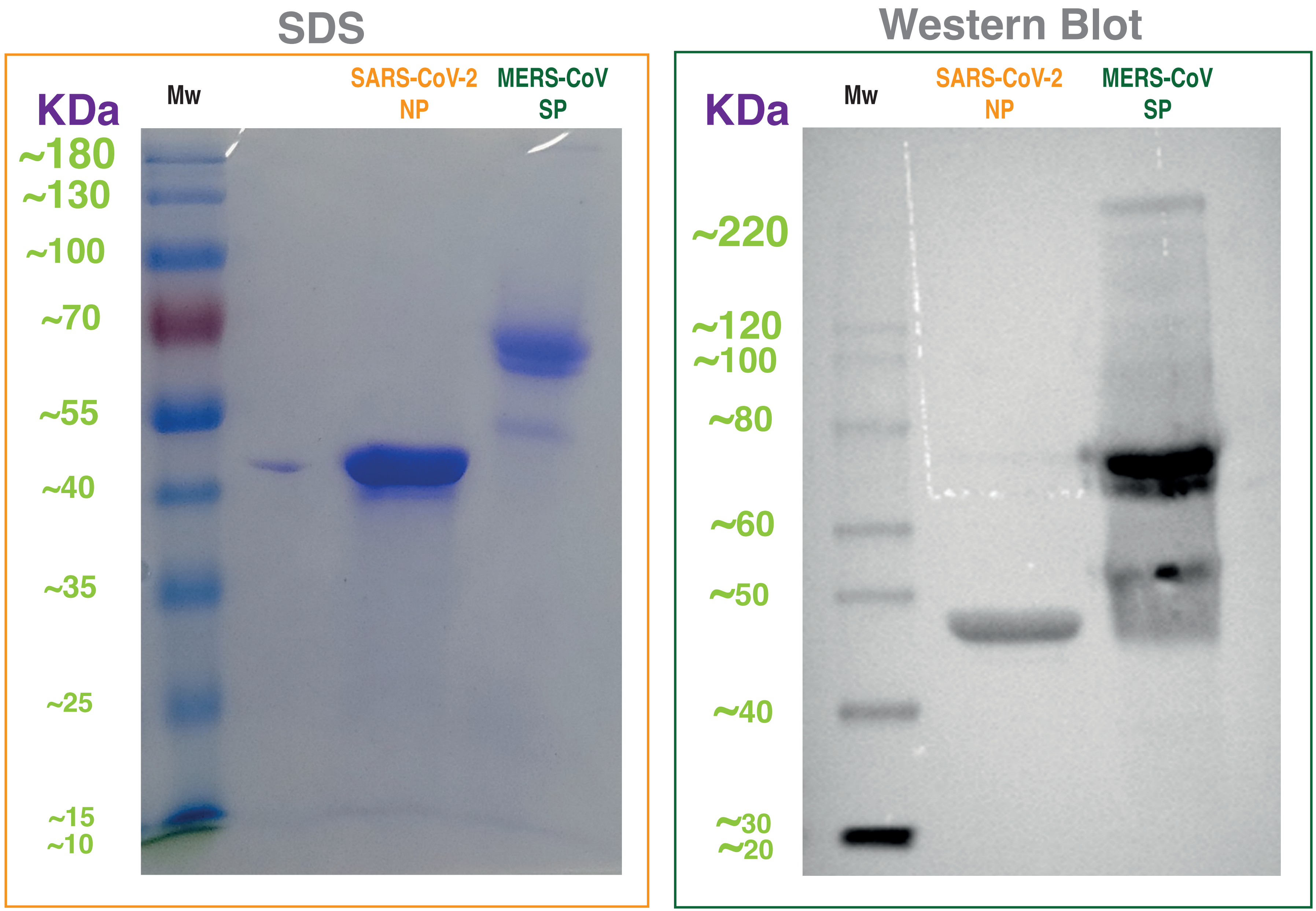
SDS
KDa
Mw
SARS-CoV-2
NP
MERS-CoV
SP
~180
~130
~100
~70
~55
~40
~35
~25
~15
~10
Western Blot
KDa
Mw
SARS-CoV-2
NP
MERS-CoV
SP
~220
~120
~100
~80
~60
~50
~40
~30
~20
