# Supplementary material for: Cost‐Effective and Time‐Saving Multiplex‐Beads Immunoassay Method to Detect Humoral Immune Responses Against Respiratory Infections
Source: J Immunol Res. 2026 Aug 26;2026:8000849. doi: 10.1155/jimr/8000849 (PMC13507927; doi:10.1155/jimr/8000849)
Supplement: Supplementary file 3 [file JIMR-2026-8000849-s003.pdf]

a)

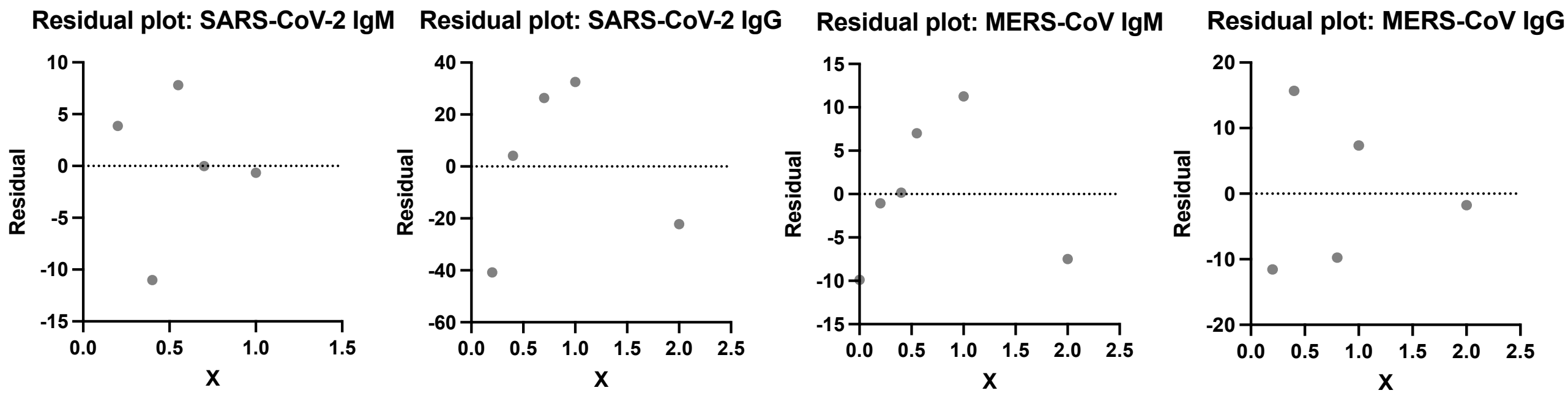
Residual plot: SARS-CoV-2 IgM
Residual
X
Residual plot: SARS-CoV-2 IgG
Residual
X
Residual plot: MERS-CoV IgM
Residual
X
Residual plot: MERS-CoV IgG
Residual
X

b)

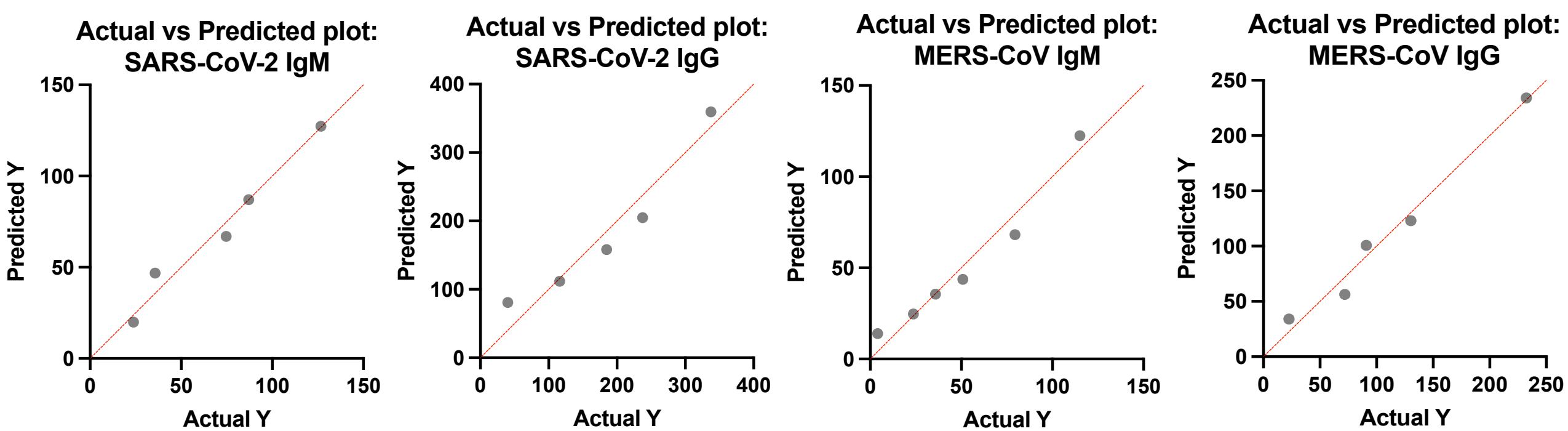
Actual vs Predicted plot: SARS-CoV-2 IgM
Predicted Y
Actual Y
Actual vs Predicted plot: SARS-CoV-2 IgG
Predicted Y
Actual Y
Actual vs Predicted plot: MERS-CoV IgM
Predicted Y
Actual Y
Actual vs Predicted plot: MERS-CoV IgG
Predicted Y
Actual Y
